# Parenting styles and Internet addiction in Chinese primary school students: a moderated sequential mediation model of self-control, rejection sensitivity, and achievement motivation

**DOI:** 10.3389/fpsyg.2026.1742553

**Published:** 2026-01-29

**Authors:** Wang Liu, Jie Huang, Yi-Ke Mo, Jia-Xin Guo, Xi Yang, Qi Wu

**Affiliations:** 1 Department of Psychology, Hunan First Normal University, Changsha, China; 2 Hunan Key Laboratory of Children’s Psychological Development and Brain, Changsha, China; 3 Department of Psychology, School of Educational Science, Hunan Normal University, Changsha, China; 4 Cognition and Human Behavior Key Laboratory of Hunan Province, Hunan Normal University, Changsha, China

**Keywords:** Internet addiction, Baumrind’s parenting styles, rejection sensitivity, self-control, achievement motivation, Chinese children

## Abstract

**Background:**

Internet addiction has reached an alarming level in Chinese children, yet the mechanisms linking parenting styles to Internet addiction remain unclear in the Chinese context characterized by intense academic competition and parental authority.

**Objective:**

This study examined how Baumrind’s parenting styles—conceptualized as a culturally meaningful framework in Chinese contexts where high parental demands coexist with varying levels of warmth—influence children’s Internet addiction through sequential mediation of self-control and rejection sensitivity, with achievement motivation—particularly salient during primary school years when motivational orientations develop—as a moderator. Baumrind’s parenting style framework provides a culturally meaningful lens for examining parenting patterns in Chinese families, particularly in contexts characterized by high parental demands.

**Methods:**

This cross-sectional study included a sample of 661 grade 4–6 children (M_age_ = 11.23 years) from central China who completed measures of parenting styles, self-control, rejection sensitivity, achievement motivation, and Internet addiction. The moderated sequential mediation model was tested using PROCESS Model 88.

**Results:**

Authoritative parenting negatively predicted Internet addiction, whereas authoritarian parenting positively predicted it. Permissive parenting showed no direct effect but significantly reduced self-control. Self-control acted as an independent mediator and enabled rejection sensitivity to serve as a subsequent mediator across all parenting styles. Achievement motivation moderated the self-control → Internet addiction path (enhancing protection) and the rejection sensitivity → Internet addiction path (buffering risk). The self-control mediation pathway showed opposite moderation patterns: positive for authoritative parenting but negative for authoritarian and permissive parenting.

**Conclusion:**

These findings challenge traditional authoritarian practices and highlight the benefits of authoritative parenting that integrates *guan* (discipline) with warmth. They underscore the need for multi-level interventions combining parent education with programs fostering children’s self-control and achievement motivation in culturally grounded family contexts.

## Highlights

- Integrates family systems theory, developmental cascade theory, and achievement goal theory through a moderated sequential mediation model, bridging traditional Chinese parenting values (*guan*) with contemporary psychological frameworks to explain Internet addiction development.
- Reveals that authoritative parenting combining high demandingness and warmth protects against Internet addiction, whereas authoritarian parenting increases risk and permissive parenting undermines self-control, underscoring the joint importance of warmth and guidance.
- Extends developmental cascade theory by identifying self-control as the core protective mechanism reducing rejection sensitivity, with intervention priorities differing across parenting styles.
- Demonstrates that achievement motivation strengthens the protective effect of self-control across parenting styles but provides limited buffering once rejection sensitivity develops, highlighting the importance of early intervention.

## 1 Introduction

Internet addiction (IA), characterized by an inability to control online impulses despite adverse consequences (Young, 1998), shows concerning prevalence rates of 10.3–23.7% among Chinese youth (Zheng et al., 2025; Xu et al., 2020) and threatens physical and mental health through depression, reduced motivation, and impaired learning (Pan et al., 2024). Given that Internet penetration among Chinese primary school students reached 95.1% in 2023 [China Internet Network Information Center (CNNIC), 2023], identifying underlying risk and protective factors for IA has become increasingly urgent.

The family environment—the primary developmental context during school-age years—shapes children’s behavioral norms and psychological development (Cox and Paley, 1997), making parenting practices a key influence on IA etiology.

Baumrind’s (1971) framework, which classifies parenting into authoritative, authoritarian, and permissive styles based on demandingness and responsiveness, provides a useful lens for examining parenting in the Chinese cultural context. In China, parenting encompasses not only parental attitudes but also expectations and demands. Parental demandingness is particularly salient due to several cultural and structural factors, including highly competitive educational environments, collectivist values emphasizing social comparison and family honor—captured in the saying “望子成龙” (hoping children become dragons; Chao, 1994)—and the belief that enduring hardship fosters success (Zeng, 2025).

These cultural patterns suggest Chinese parents commonly maintain high demands whether adopting warm or strict approaches. Both authoritative parenting (high demands combined with high responsiveness) and authoritarian parenting (high demands combined with low responsiveness and strict discipline) emphasize strong regulation of children’s behavior, differing primarily in emotional warmth. In contrast, permissive parenting, characterized by low demands and limited guidance, tends to emerge in Chinese contexts through distinct pathways, such as reduced parental supervision among “left-behind children” due to labor migration, or ideological shifts among some families influenced by Western views that prioritize autonomy and independence (Chen et al., 2021).

Previous research demonstrates that positive parenting practices are associated with lower IA risk (Vossen et al., 2024; Guo et al., 2024), whereas negative parenting practices are linked to elevated IA vulnerability (Doo and Kim, 2022). However, findings regarding the effects of specific parenting styles remain inconsistent across cultural contexts. For instance, authoritative parenting has been identified as a protective factor in some studies (Varma et al., 2018), whereas other research reports nonsignificant associations (Setiawati et al., 2021). Likewise, the effects of authoritarian (Setiawati et al., 2021) and permissive parenting (Fatemisough et al., 2024; Anandari, 2016) on IA appear culturally contingent, with evidence of both risk-enhancing and neutral effects across different populations. More importantly, relatively few studies have systematically examined the psychological mechanisms through which different parenting styles influence IA vulnerability in children in Chinese context, or the boundary conditions under which these mechanisms operate.

**Figure 1:**
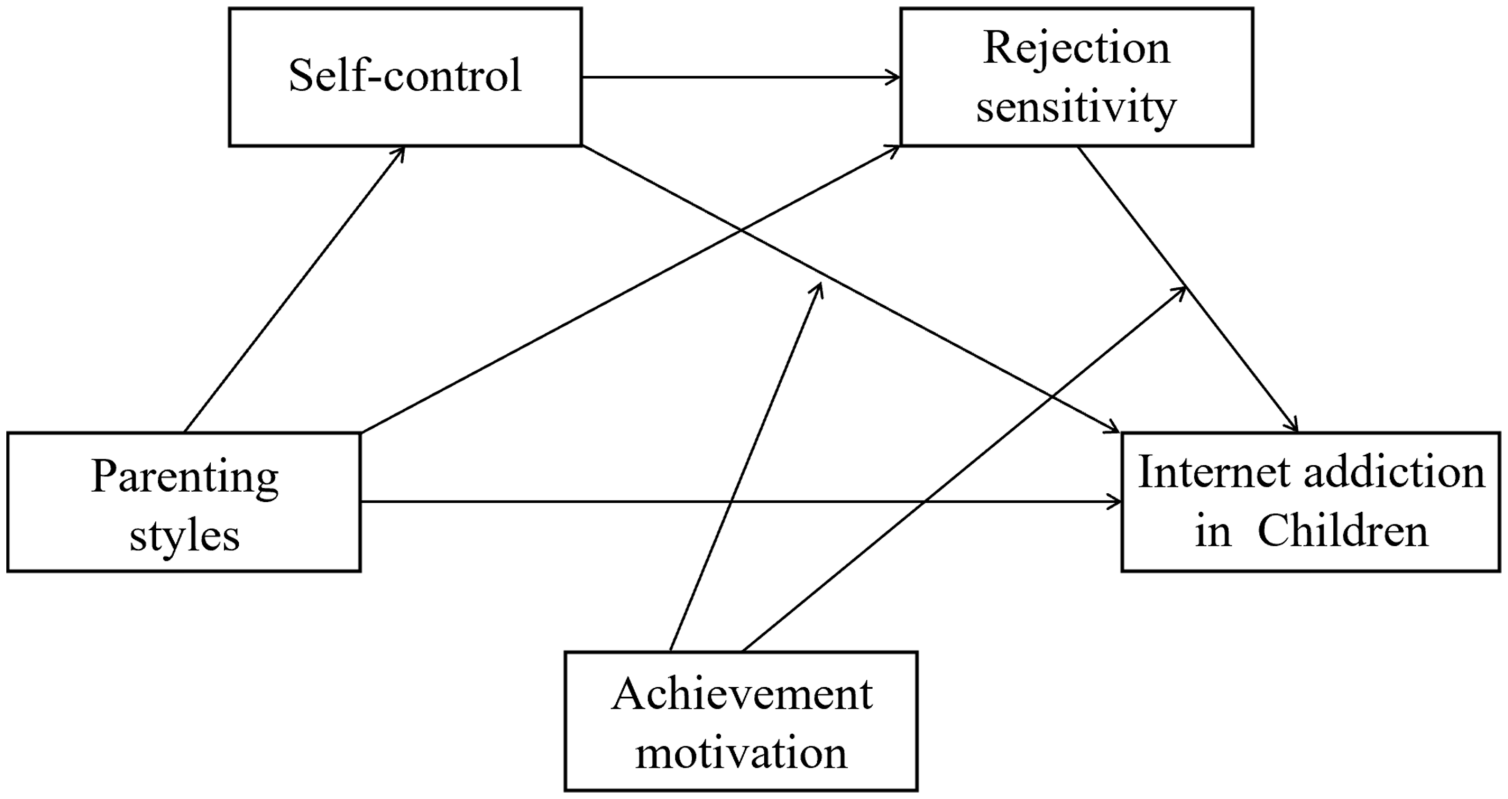
The proposed moderated sequential mediation model of the relationship between parenting styles and Internet addiction in Chinese children. Achievement motivation (W) was modeled as a moderator. Its direct effect on Internet addiction (Y) was estimated for statistical completeness, but no direct W → Y path was hypothesized and is therefore not shown in the conceptual model.

To address these gaps, the present study proposes an integrated moderated sequential mediation model (see Figure 1) to examine how Baumrind’s parenting styles influence IA among Chinese primary school students. Drawing on developmental cascade models (Masten and Cicchetti, 2010) and compensatory Internet use theory (Kardefelt-Winther, 2014), we hypothesize that parenting practices initially shape children’s self-control capacities, which subsequently influence rejection sensitivity, ultimately contributing to IA risk through compensatory Internet use. Furthermore, grounded in Achievement Goal Theory (Dweck and Leggett, 1988), we examine achievement motivation as a key individual difference that moderates these pathways, thereby identifying boundary conditions under which parenting-related risks are more or less likely to translate into IA.

### 1.1 Direct effects of Baumrind’s parenting styles on Internet addiction

Regarding direct effects, authoritative parenting provides clear behavioral standards alongside emotional support, creating secure environments where children develop adaptive coping strategies (Williams et al., 2009; Karaer and Akdemir, 2019), and may predict IA negatively (H1a). Authoritarian parenting creates emotional deficits that may be consistent with compensatory Internet use according to the compensatory Internet use theory (Kardefelt-Winther, 2014; Lei and Wu, 2007; Xu et al., 2014), while excessive control may paradoxically increase prohibited behaviors through psychological reactance based on psychological reactance theory (Brehm, 1966; Van Petegem et al., 2015), may predict IA positively (H1b). Permissive parenting may leave children with limited guidance to regulate Internet use (Eastin et al., 2006; van den Eijnden et al., 2010), which in turn may also predict IA positively (H1c).

### 1.2 The mediating roles of self-control

Parenting styles may influence Internet addiction (IA) indirectly through psychological mechanisms, particularly self-control—the capacity to regulate behavior, thoughts, and emotions (Tangney et al., 2004). Low self-control increases vulnerability to impulsive behaviors, including excessive Internet use (Baumeister et al., 2007; Hofmann et al., 2012), with robust evidence linking self-control deficits to IA among East Asian students (Li et al., 2021).

Importantly, parenting styles differentially shape self-control development. Authoritative parents help children internalize behavioral standards and develop impulse control through reasoning rather than punishment (Baumrind, 1991; Steinberg, 2001), strengthening self-regulatory capacities. Conversely, authoritarian parenting may undermine self-control by fostering external regulatory orientation wherein children rely on parental punishment and surveillance rather than internal self-regulation (Grolnick and Ryan, 1989); when supervision is absent, they may lack internal resources to resist impulsive behaviors (Larose et al., 2003). Similarly, permissive parenting associates with higher impulsivity and poorer self-regulation (Pinquart and Gerke, 2019; Piotrowski et al., 2013) as children rarely receive explicit instruction in impulse regulation or adequate self-regulatory models. Therefore, we hypothesize that self-control mediates the relationships between all three parenting styles and IA (H2a–c).

### 1.3 The mediating roles of rejection sensitivity

Rejection sensitivity—anxious anticipation and overreaction to rejection cues (Downey and Feldman, 1996)—is identified as a significant IA risk factor (Tao et al., 2022; Fan et al., 2022; Xu et al., 2025), as rejection-sensitive individuals seek relief through excessive Internet use and prefer virtual environments where rejection cues are less salient (Kardefelt-Winther, 2014).

Parenting styles differentially influence rejection sensitivity development. Authoritative parents provide substantial emotional support and rarely reject children, weakening formation of “rejection–anxiety” associations (Downey and Feldman, 1996). The emphasis on reasoning and guided autonomy helps children develop effective emotion regulation strategies when facing interpersonal challenges (Bernier et al., 2010), reducing their tendency to catastrophize potential rejection scenarios. In contrast, authoritarian environments with harsh discipline, rejection, and coldness contribute to rejection sensitivity development (Downey et al., 2004); longitudinal research demonstrates that parental psychological control and coercion positively predict rejection sensitivity in early adolescents (Rowe et al., 2015). Children experience frequent negative encounters involving criticism and conditional acceptance, fostering anticipatory anxiety and defensive overreaction to rejection cues. Permissive parenting, however, involves low conflict and infrequent explicit rejection, lacking the critical rejection-related experiences that heighten rejection sensitivity. Given that permissive parenting’s influence likely operates through alternative mechanisms such as poor self-regulation (Steinberg, 2001), we hypothesize that rejection sensitivity mediates the relationships between authoritative parenting (H3a) and authoritarian parenting (H3b) and IA, but not the relationship between permissive parenting and IA (H3c).

### 1.4 Sequential mediation through self-control and rejection sensitivity

Building on developmental cascade models (Masten and Cicchetti, 2010), we propose that self-control and rejection sensitivity operate sequentially in linking parenting to IA. Low self-control reduces children’s ability to regulate emotions and impulses, increasing susceptibility to anxiety-driven interpretations of social situations and heightened rejection sensitivity (Blair and Raver, 2015; Fan et al., 2022). Heightened rejection sensitivity, in turn, motivates excessive Internet use as children seek to avoid interpersonal risks (Kardefelt-Winther, 2014; Tao et al., 2022). Accordingly, we hypothesize that all three parenting styles influence IA through this sequential pathway: authoritative parenting reduces IA via higher self-control and lower rejection sensitivity, whereas authoritarian and permissive parenting increase IA via lower self-control and higher rejection sensitivity (H4a–c).

### 1.5 The moderating role of achievement motivation

While parenting and mediating mechanisms explain how IA risk develops, individual differences may determine whether these risks materialize. Achievement motivation—an individual’s drive to pursue success and master challenging tasks (Martin, 2012)—may moderate the effects of self-control and rejection sensitivity on IA. Drawing on Achievement Goal Theory (Dweck and Leggett, 1988; Elliot, 1999), we propose that achievement motivation serves as a boundary condition, particularly in Chinese primary school contexts where academic achievement is highly emphasized (Chao, 1994) and motivational patterns are forming (Wigfield et al., 2015; Gottfried et al., 2001). High achievement motivation may promote goal-directed regulation and buffer against self-regulatory deficits and emotional distress (Duckworth et al., 2007; Hofer and Fries, 2016).

For the self-control pathway, children with high achievement motivation may rely on future-oriented goals to regulate Internet use, reducing dependence on trait self-control. Thus, even children with lower self-control may restrain excessive use, weakening the protective effect of self-control at high motivation levels. Conversely, when achievement motivation is low, self-control is more critical for regulating Internet use (Fujita, 2011; Inzlicht and Schmeichel, 2012; Milyavskaya et al., 2015). Therefore, achievement motivation is hypothesized to moderate the self-control–IA link (H5).

For the rejection sensitivity pathway, high achievement motivation provides adaptive coping and a future-oriented sense of competence, reducing reliance on Internet use to manage social stress and rejection-related anxiety (Dweck, 2006; Burnette et al., 2013; Duckworth et al., 2007). In contrast, children with high rejection sensitivity and low achievement motivation face a “double vulnerability,” heightening IA risk. Thus, achievement motivation is expected to moderate the rejection sensitivity–IA link, weakening its effect at higher levels (H6).

### 1.6 The present study

The present study investigates the associations between Baumrind’s three parenting styles and IA among Chinese primary school students using an integrated moderated sequential mediation framework (see Figure 1). Specifically, this framework advances existing literature by: (1) providing culturally grounded evidence on the direct effects of parenting styles on IA (H1a–c); (2) identifying self-control (H2a–c) and rejection sensitivity (H3a–c) as distinct yet complementary mediating mechanisms; (3) testing a novel sequential mediation pathway that specifies the temporal ordering of these psychological processes (H4a–c); and (4) examining achievement motivation as a moderator that delineates critical boundary conditions (H5, H6). By clarifying the ordered psychological mechanisms linking parenting to IA within the Chinese cultural context, this integrative framework advances theory and informs multi-level interventions, including family-based programs targeting parenting practices, individual-focused strategies to strengthen self-regulation, and school-based initiatives that cultivate adaptive achievement motivation.

## 2 Materials and methods

### 2.1 Participants and procedure

A total of 721 grade 4–6 students were recruited from selected classes in two public primary schools in Changsha, Hunan Province, using a convenience sampling method, targeting classes that were readily accessible. Within the selected classes, all students whose parents provided consent were invited to participate. The researchers reviewed all completed questionnaires for data quality. Responses were excluded if more than 10 items were missing across all questionnaires, if more than two consecutive scenarios (six items) were missing in the rejection sensitivity scale, or if identical response options were selected across all items. After applying these criteria, 661 valid responses remained, yielding a valid response rate of 91.68%. The mean age of the participants was 11.23 years (SD = 0.77). The gender distribution was nearly balanced, with 49.5% boys and 50.5% girls. Of the 661 participants, 4.4% were in grade 4, 50.7% in grade 5, and 44.9% in grade 6. Additionally, 65.2% of the participants reported having siblings, and 75.6% resided in urban areas. Regarding parental education, 31.8% of fathers held a bachelor’s degree, which was the same as that (31.8%) observed for mothers. A one-way ANOVA indicated that IA levels significantly differed across groups based on sibling status (*F* = 5.70, *p* < 0.01) and family residence (*F* = 3.70, *p* < 0.05), suggesting that these demographic factors may influence IA. Therefore, these two sociodemographic variables were included as covariates in the subsequent analyses to control for potential confounding effects. Additional demographic information is presented in Table 1.

**Table 1:** Demographic profile of the participants (*N* = 661).

Variable	Internet addiction (M ± SD)	*N* (%)	*t/F*	*p*-value
General characteristics of children with Internet addiction				
Gender			1.89	0.06
Boys	37.25 ± 10.75	327 (49.5)		
Girls	35.58 ± 11.86	334 (50.5)		
Grade			2.29	0.07
The fourth grade	33.59 ± 9.53	29 (4.4)		
The fifth grade	35.84 ± 10.74	335 (50.7)		
The sixth grade	37.32 ± 12.10	297 (44.9)		
Only child			5.70	0.004
Yes	37.43 ± 12.06	189 (28.6)		
No	34.86 ± 9.54	431 (65.2)		
Missing	32.76 ± 9.83	41 (6.2)		
Home location			3.70	0.03
City	35.77 ± 10.67	500 (75.6)		
Countryside	38.19 ± 12.93	155 (23.5)		
Missing	42.83 ± 17.42	6 (0.9)		
Paternal education level			0.60	0.62
High school and below	37.09 ± 11.17	206 (31.2)		
Bachelor’s degree	35.97 ± 10.81	210 (31.8)		
Above bachelor’s degree	35.82 ± 11.65	172 (26.0)		
Missing	37.10 ± 12.64	73 (11.0)		
Maternal education level			0.89	0.44
High school and below	37.36 ± 11.17	209 (31.6)		
Bachelor’s degree	36.11 ± 11.04	210 (31.8)		
Above bachelor’s degree	35.51 ± 11.48	166 (25.1)		
Missing	36.57 ± 12.36	76 (11.5)		

To determine the appropriate sample size, we conducted a Monte Carlo power analysis for indirect effects (Schoemann et al., 2017). Results indicated that a minimum of 205 participants would be required to achieve a statistical power of 90% in a serial mediation model. Therefore, the current sample size was sufficient for robust statistical analysis.

This study received ethical approval from the Ethics Committee of Science and Technology at our university and adhered to the principles outlined in the Declaration of Helsinki. Data were collected between February and May 2023. Prior to participation, written informed consent was obtained from both the children and their parents. Participants were asked to complete a structured questionnaire that assessed demographic characteristics, parenting styles, self-control, rejection sensitivity, achievement motivation, and IA. They were instructed to finish the questionnaire within 40 min and were allowed to seek clarification from a trained assistant teacher if needed. Participants were informed of their right to withdraw at any point prior to submitting the completed questionnaire. The researchers reviewed the completed questionnaires for data quality, and responses that did not meet the predefined exclusion criteria were excluded from the analysis.

### 2.2 Measurements

#### 2.2.1 Parental authority questionnaire

The Parental Authority Questionnaire (PAQ; Buri, 1991), developed based on Baumrind’s (1971) typology of parenting styles—authoritarian, authoritative, and permissive—adopts a classification approach that is also familiar to Chinese parents. The PAQ consists of three subscales, each containing 10 items, for a total of 30 items. Participants rate their parents’ parenting behaviors on a 5-point Likert scale (1 = Strongly Disagree, 5 = Strongly Agree). The subscales include: authoritative (Items 4, 5, 8, 11, 15, 20, 22, 23, 27, 30), authoritarian (Items 2, 3, 7, 9, 12, 16, 18, 25, 26, 29), and permissive (Items 1, 6, 10, 13, 14, 17, 19, 21, 24, 28). Scores for each parenting style range from 10 to 50, obtained by summing the respective items, with higher scores indicating greater endorsement of that parenting style. Children rated their parents’ parenting behaviors as a whole rather than evaluating mothers and fathers separately, reflecting children’s overall perception of family parenting practices.

For the present study, the Parental Authority Questionnaire (PAQ) was translated and adapted into Chinese and demonstrated acceptable reliability and validity. The Cronbach’s alpha coefficients were 0.80 for the total scale, 0.88 for the authoritative subscale, 0.83 for the authoritarian subscale, and 0.69 for the permissive subscale. The McDonald’s omega coefficients were 0.87 for the total scale and 0.88, 0.83, and 0.70 for the authoritative, authoritarian, and permissive subscales, respectively. Although the permissive parenting subscale showed borderline internal consistency, its omega value met the conventional threshold for acceptable reliability. The confirmatory factor analysis (CFA) indicated an acceptable model fit, with a chi-square to degrees of freedom ratio (*χ*^2^/df) of 2.65, a Root Mean Square Error of Approximation (RMSEA) of 0.05, a Standardized Root Mean Square Residual (SRMR) of 0.08, a Comparative Fit Index (CFI) of 0.89, and a Tucker–Lewis Index (TLI) of 0.88. The CFA of the authoritative subscale indicated a good model fit (*χ*^2^/df = 2.70, RMSEA = 0.05, SRMR = 0.03, CFI = 0.97, TLI = 0.95). The CFA of the authoritarian subscale indicated a good model fit (*χ*^2^/df = 3.16, RMSEA = 0.06, SRMR = 0.04, CFI = 0.94, TLI = 0.91). The CFA of the permissive subscale indicated an acceptable model fit (*χ*^2^/df = 3.03, RMSEA = 0.06, SRMR = 0.05, CFI = 0.91, TLI = 0.87).

#### 2.2.2 Grasmick self-control scale

The Self-Control Scale (Grasmick et al., 1993) consists of 24 items divided into six dimensions: impulsivity, preference for simple rather than complex tasks, risk seeking, preference for physical rather than cerebral activities, self-centered orientation, and a volatile temper associated with low frustration tolerance. Each item is rated on a 4-point scale (1 = Strongly Agree, 4 = Strongly Disagree), with higher scores indicating higher self-control. In this study, the Cronbach’s *α* coefficient for the scale was 0.91. The CFA results showed that this scale had an acceptable model fit (*χ*^2^/df = 3.46, RMSEA = 0.06, SRMR = 0.06, CFI = 0.84, TLI = 0.82).

#### 2.2.3 Children’s rejection sensitivity questionnaire

The Children’s Rejection Sensitivity Questionnaire (CRSQ), originally developed by Downey et al. (1998), is designed to measure children’s tendencies to respond to potential rejection in typical social interactions. The original version contains 12 hypothetical scenarios, each followed by three items assessing anxiety expectations, anger expectations, and perceived likelihood of rejection. Responses are rated on a 6-point Likert scale, with higher scores indicating greater rejection sensitivity. Nesdale et al. (2014) suggested that, when studying rejection sensitivity in primary school children, only the six scenarios involving interactions with teachers and classmates from the original 12 should be retained, as these are more age-appropriate. Building on this adaptation, Ding et al. (2018) revised and translated the CRSQ into Chinese, producing a version that demonstrated good reliability and validity for use with Chinese primary school students. The Cronbach’s alpha coefficient for the CRSQ was 0.84. The CFA results showed that this scale had an acceptable model fit (*χ*^2^/df = 3.86, RMSEA = 0.07, SRMR = 0.07, CFI = 0.87, TLI = 0.84).

#### 2.2.4 Achievement motive scale

The Achievement Motivation Scale (AMS; Gjesme and Nygard, 1970; Chinese version by Ye and Hai, 1992) consists of 30 items, equally divided into two subscales: success motivation (15 items) and failure avoidance (15 items). Each item is rated on a 4-point scale (1 = completely true, 4 = completely untrue). The total achievement motive score is obtained by subtracting the total score of the failure avoidance subscale from that of the success motivation subscale, with higher scores indicating a stronger achievement motive. In this study, the Cronbach’s *α* coefficient for the AMS was 0.86. The CFA results showed that the scale had a good model fit (*χ*^2^/df = 2.89, RMSEA = 0.05, SRMR = 0.06, CFI = 0.89, TLI = 0.88).

#### 2.2.5 Internet addiction test

IA was assessed using the Internet Addiction Test (IAT) developed by Young (1999). The scale included 20 items rated on a 5-point Likert scale (1 = none, 5 = always). Total scores ranged from 20 to 100, with higher scores indicating a greater risk of IA. The scale has been validated in primary school students, demonstrating good reliability and validity (Liu W. et al., 2025). The Cronbach’s alpha coefficient for the scale was 0.880 in this study. The CFA results showed that the scale had a good model fit (*χ*^2^/df = 2.82, RMSEA = 0.05, SRMR = 0.04, CFI = 0.92, TLI = 0.90).

### 2.3 Statistical analysis

All statistical analyses were performed using IBM SPSS Statistics version 22.0 and Mplus version 8.3. Descriptive and inferential analyses were conducted to examine participants’ gender, grade level, only-child status, place of residence, paternal education level, and maternal education level. Independent-samples *t*-tests were used to assess gender differences in IA. One-way analyses of variance (ANOVAs) were applied to examine differences in IA across grade level, only-child status, place of residence, paternal education level, and maternal education level. Pearson’s correlation analysis was performed to examine the relationships among authoritarian, authoritative, and permissive parenting styles, rejection sensitivity, self-control, achievement motivation, and IA. Confirmatory factor analysis (CFA) was performed in Mplus 8.3 to evaluate the construct validity of the scales. Harman’s single-factor test was employed to assess potential common method bias. Missing data were handled using listwise deletion, consistent with the default procedure in PROCESS. Model 88 of the PROCESS was employed to test the moderated sequential mediation model. The model examined authoritative parenting style, authoritarian parenting style, permissive parenting style, as the predictor, with self-control and rejection sensitivity as serial mediators, achievement motivation as the moderator, and IA as the outcome. All continuous variables were mean-centered prior to analysis to facilitate interpretation of interaction terms and reduce multicollinearity (Aiken and West, 1991). Indirect effects were estimated using 5,000 bootstrap resamples, and statistical significance was determined based on 95% bias-corrected confidence intervals that did not include zero.

## 3 Results

### 3.1 Common method variance

Harman’s (1967) single-factor test was conducted to assess common method bias. Results indicated that 28 factors had eigenvalues greater than 1, with the largest factor accounting for 11.66% of the variance, well below the conventional 40% threshold. While these results suggest that no single factor dominated, all variables were collected via child self-report in a single session, and other sources of method bias cannot be entirely ruled out. To reduce potential common method bias, several procedural safeguards were implemented, including assurances of confidentiality, standardized instructions, and exclusion of questionnaires exhibiting inattentive or uniform response patterns.

### 3.2 Preliminary correlation analyses

Pearson correlation analyses revealed that authoritative parenting was negatively correlated with IA (*r* = −0.13, *p* < 0.001) and rejection sensitivity (*r* = −0.14, *p* < 0.001), and positively correlated with self-control (*r* = 0.17, *p* < 0.001) and achievement motivation (*r* = 0.13, *p* < 0.001). Authoritarian parenting was positively correlated with IA (*r* = 0.14, *p* < 0.001) and rejection sensitivity (*r* = 0.13, *p* < 0.001), negatively correlated with self-control (*r* = −0.26, *p* < 0.001), and not significantly correlated with achievement motivation (*r* = −0.02, *p* > 0.05). Permissive parenting was not significantly correlated with IA. Additionally, self-control was negatively correlated with rejection sensitivity (*r* = −0.16, *p* < 0.001), and achievement motivation was negatively correlated with IA (*r* = −0.27, *p* < 0.001) (see Table 2). Overall, these are small associations.

**Table 2:** Means, standard deviations, and correlations of all variables (*N* = 661).

Variables	1	2	3	4	5	6	7
1. Authoritative style	—						
2. Authoritarian style	−0.28***	—					
3. Permissive style	0.43***	0.14***	—				
4. Rejection sensitivity	−0.14***	0.13***	−0.05	—			
5. Self-control	0.17***	−0.26***	−0.13***	−0.16***	—		
6. Achievement motivation	0.13***	−0.02	0.05	−0.29***	0.17***	—	
7. Internet addiction	−0.13***	0.14***	0.001	0.32***	−0.28***	−0.27***	—
M	33.49	24.73	25.06	48.93	68.33	5.95	36.40
SD	7.67	6.38	5.19	14.02	12.75	13.80	11.35

***p < 0.001.

### 3.3 The moderated sequential mediation model across three parenting styles

The total effects of the three parenting styles indicated that authoritative parenting negatively predicted IA (*β* = −0.17, *p* < 0.05), whereas authoritarian parenting positively predicted IA (*β* = 0.17, *p* < 0.05); permissive parenting, however, showed a non-significant effect (*β* = 0.07, *p* > 0.05). After controlling for mediators and the moderator, the direct effects of all three parenting styles—authoritative (*β* = −0.01, *p* > 0.05), authoritarian (*β* = 0.09, *p* > 0.05), and permissive (*β* = −0.02, *p* > 0.05)—became non-significant (see Table 3). These results suggest that the associations between parenting styles and IA are primarily transmitted indirectly through self-control and rejection sensitivity.

**Table 3:** Model for regression analysis between variables (*N* = 661).

Predictor variable/path	Authoritative *B* (SE)	Authoritarian *B* (SE)	Permissive *B* (SE)	Shared value *B* (SE)
Outcome variable: self-control (M1)				
X → M1	0.34*** (0.07)	−0.35*** (0.08)	−0.49*** (0.11)	
*R^2^*				0.11
*F*				15.74***
Outcome variable: rejection sensitivity (M2)				
X → M2	−0.13 (0.09)	0.18* (0.09)	−0.13 (0.12)	
M1 → M2				−0.14** (0.04)
*R^2^*				0.05
*F*				5.63***
Outcome variable: Internet addiction (Y)				
X → Y	−0.01 (0.06)	0.09 (0.07)	−0.02 (0.09)	
M1 → Y				−0.26*** (0.04)
M2 → Y				0.22*** (0.03)
W → Y				−0.50** (0.18)
M1 × W				0.01*** (0.002)
M2 × W				−0.01* (0.002)
*R^2^*				0.22
*F*				18.11***

*B* (SE) = unstandardized coefficient with standard error in parentheses. *p < 0.05. **p < 0.01. ***p < 0.001. Shared values indicate paths where coefficients were identical across all three predictors. These include: (1) the effect of self-control (M1) on rejection sensitivity (M2) and its moderation by achievement motivation (W), and (2) all paths predicting Internet addiction (Y) from M1, M2, and W. Achievement motivation was included as a moderator; its direct effect on Internet addiction is reported in Table 3 for completeness but was not theoretically hypothesized. Model-specific paths (first three columns) show how authoritative parenting style, authoritarian parenting style, and permissive parenting style differentially predict M1, M2, and Y.

**Figure 2:**
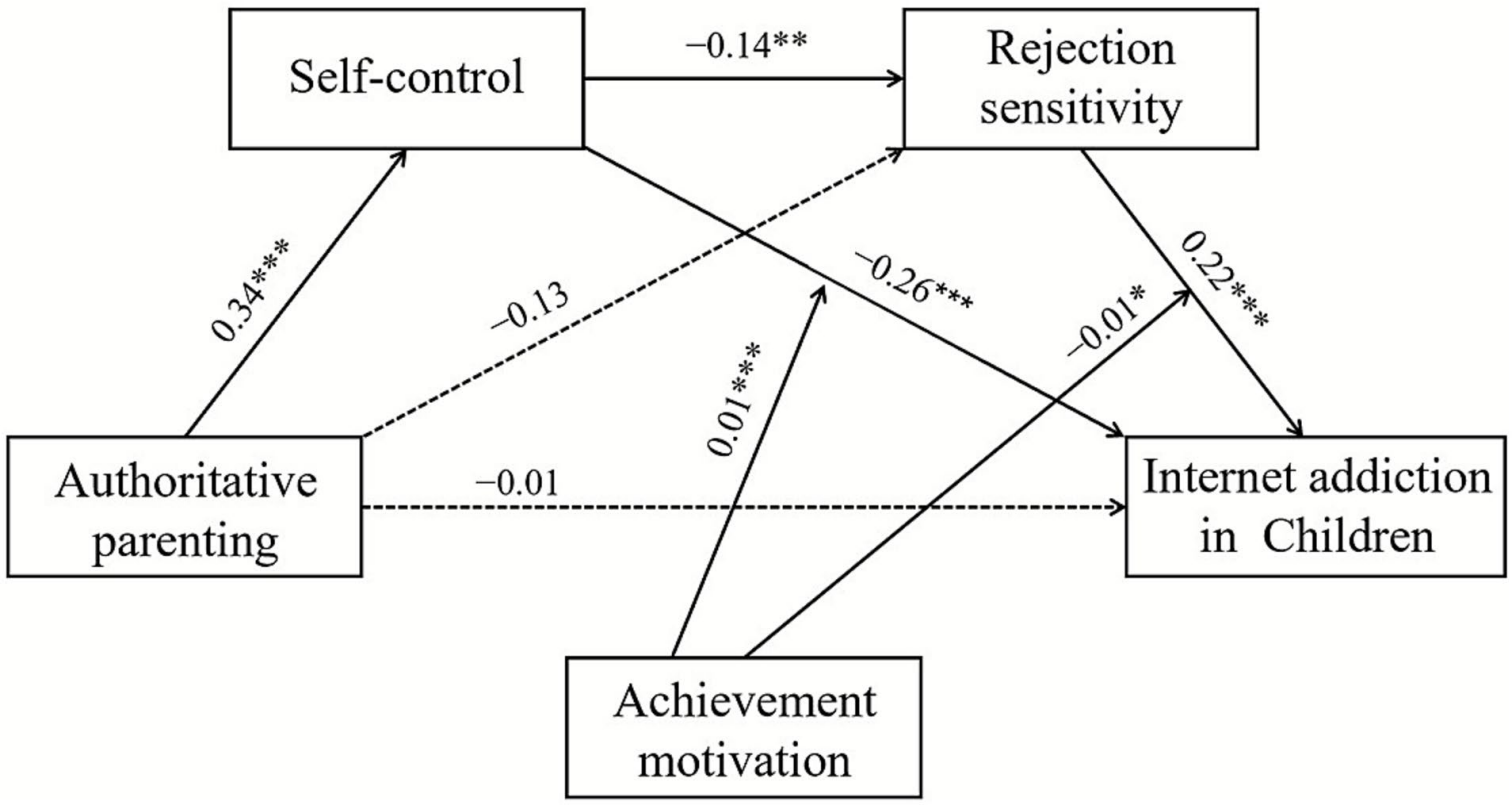
The moderated sequential-mediation model between authoritative parenting and Internet addiction. *p < 0.05, **p < 0.01, ***p < 0.001.

**Figure 3:**
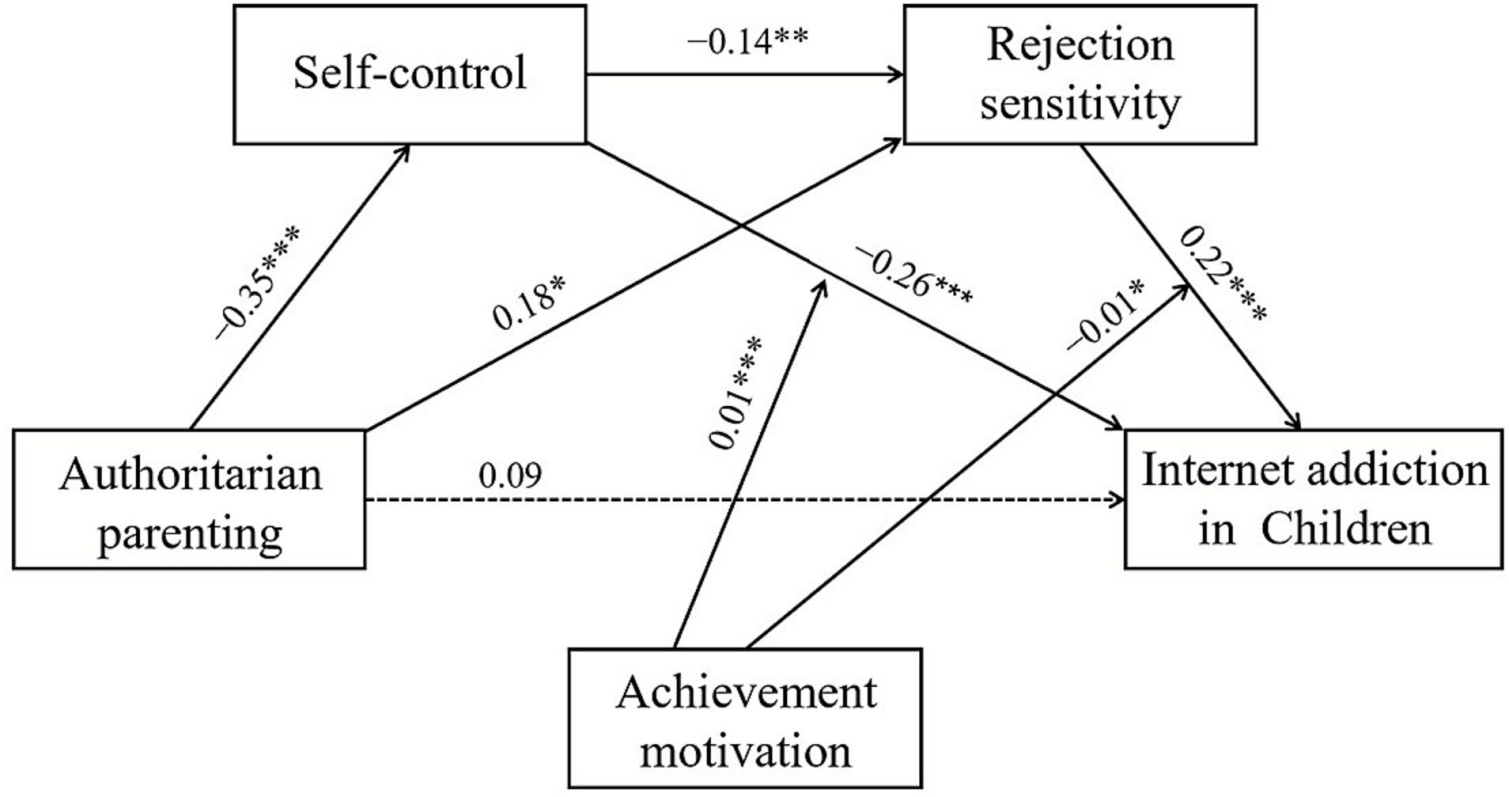
The moderated sequential-mediation model between authoritarian parenting and Internet addiction. *p < 0.05, **p < 0.01, ***p < 0.001.

**Figure 4:**
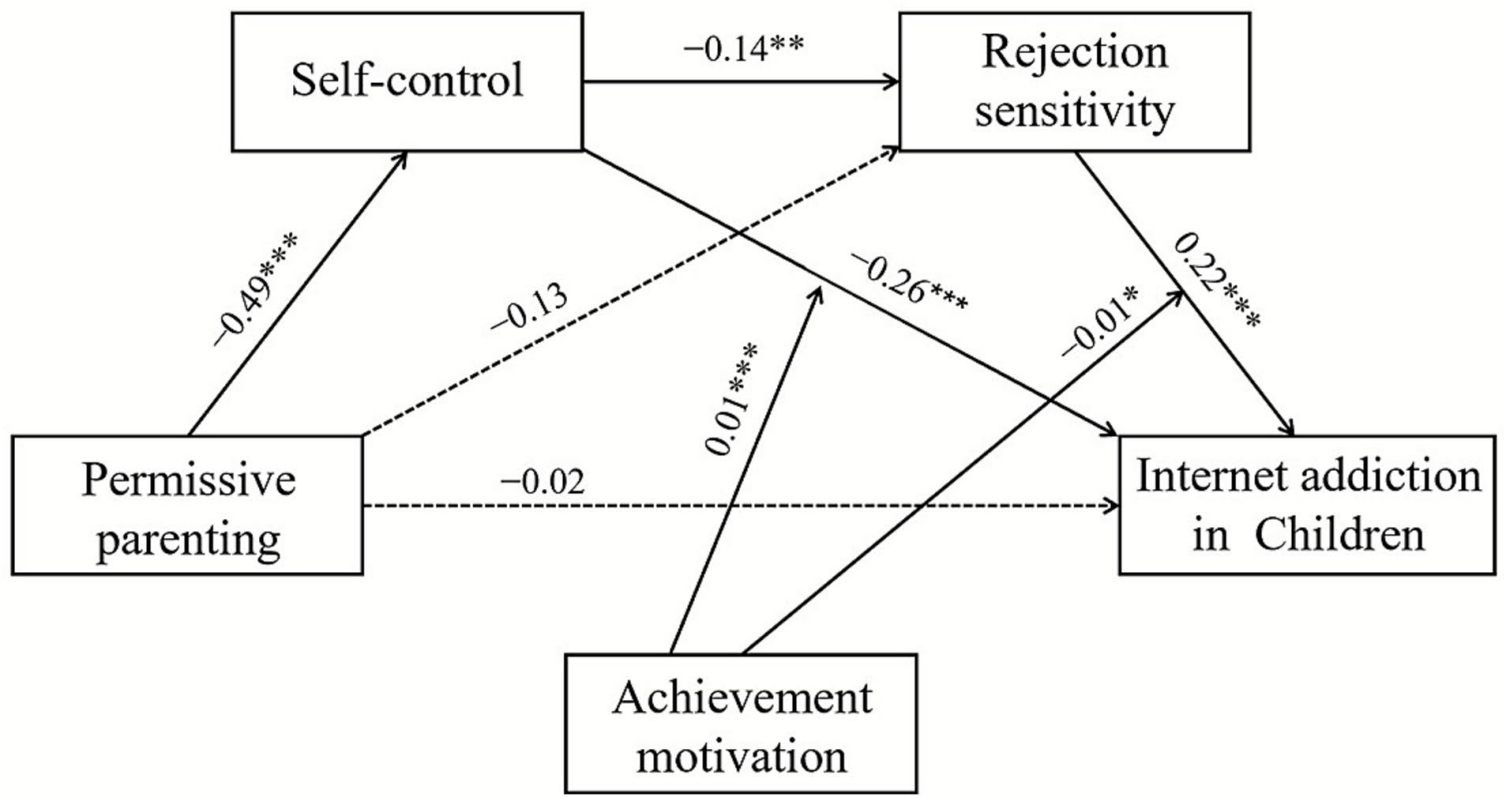
The moderated sequential-mediation model between permissive parenting and Internet addiction. *p < 0.05, **p < 0.01, ***p < 0.001.

As the direct effects of authoritative parenting style, authoritarian parenting style, and permissive parenting style, on self-control and rejection sensitivity differed in the model, the subsequent mediation chain from self-control through rejection sensitivity to IA remained structurally identical with equivalent coefficients. We established three separate models based on the three parenting styles, as shown in Figures 2–4. The varying coefficients are presented in the first three columns of Table 3, while the invariant coefficients are presented in the fourth column.

#### 3.3.1 Differential direct effects: parenting styles on mediators

As shown in the upper section of Table 3, all three parenting styles as predictors significantly influenced self-control, although with varying magnitudes. Authoritative parenting style (*β* = 0.34, *p* < 0.001; see Figure 2), authoritarian parenting style (*β* = −0.35, *p* < 0.001; see Figure 3), and permissive parenting style (*β* = −0.49, *p* < 0.001; see Figure 4). These results indicated that all three parenting styles are related to the first mediator, self-control (M1).

Regarding the direct paths to rejection sensitivity (M2), the pattern diverged. Authoritative parenting showed a non-significant direct effect (*β* = −0.13, *p* > 0.05), whereas authoritarian parenting exerted a significant positive effect on rejection sensitivity (*β* = 0.18, *p* < 0.05). Permissive parenting also exhibited a non-significant direct effect (*β* = −0.13, *p* > 0.05). This pattern suggests that authoritarian parenting influences rejection sensitivity both indirectly through reduced self-control and directly, while the effects of authoritative and permissive parenting are primarily transmitted through the indirect pathway.

#### 3.3.2 Shared mediation chain and achievement motivation as moderator

The mediation chain from self-control through rejection sensitivity to IA (Y) showed identical patterns across all three parenting models (see Table 3, rightmost column) that self-control negatively predicted rejection sensitivity (*β* = −0.14, *p* < 0.01). And both mediators significantly predicted IA. Self-control predicted IA negatively (*β* = −0.26, *p* < 0.001), and rejection sensitivity exerted an even stronger positive effect (*β* = 0.22, *p* < 0.001).

Achievement motivation also showed a main effect on IA (*β* = −0.50, *p* < 0.01). Importantly, the self-control × achievement motivation interaction (*β* = 0.01, *p* < 0.001) and the rejection sensitivity × achievement motivation interaction both were significant (*β* = −0.01, *p* < 0.05).

**Figure 5:**
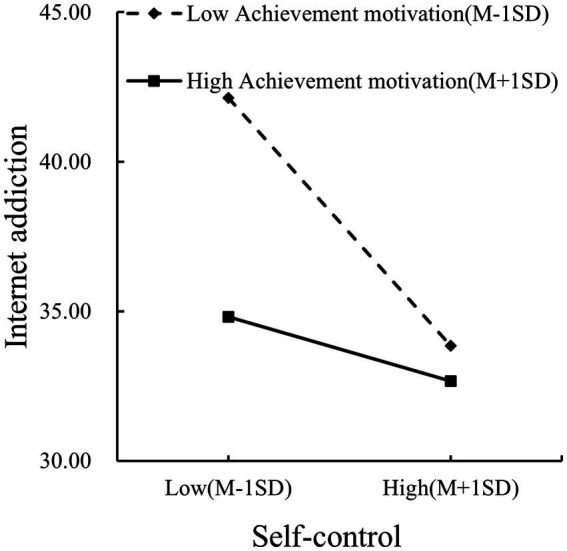
Achievement motivation moderates the self-control–Internet addiction pathway linking authoritative parenting to Internet addiction.

To further clarify the moderating effect of achievement motivation, a simple slopes analysis was conducted following the methodology proposed by Toothaker (1994) (see Figure 5). The effect of self-control on IA was significantly negative at all levels of achievement motivation, but attenuated as achievement motivation increased: low achievement motivation (index = −0.32, 95% CI [−0.42, −0.23]), mean achievement motivation (index = −0.20, 95% CI [−0.27, −0.13]), and high achievement motivation (index = −0.08, 95% CI [−0.16, −0.01]).

**Figure 6:**
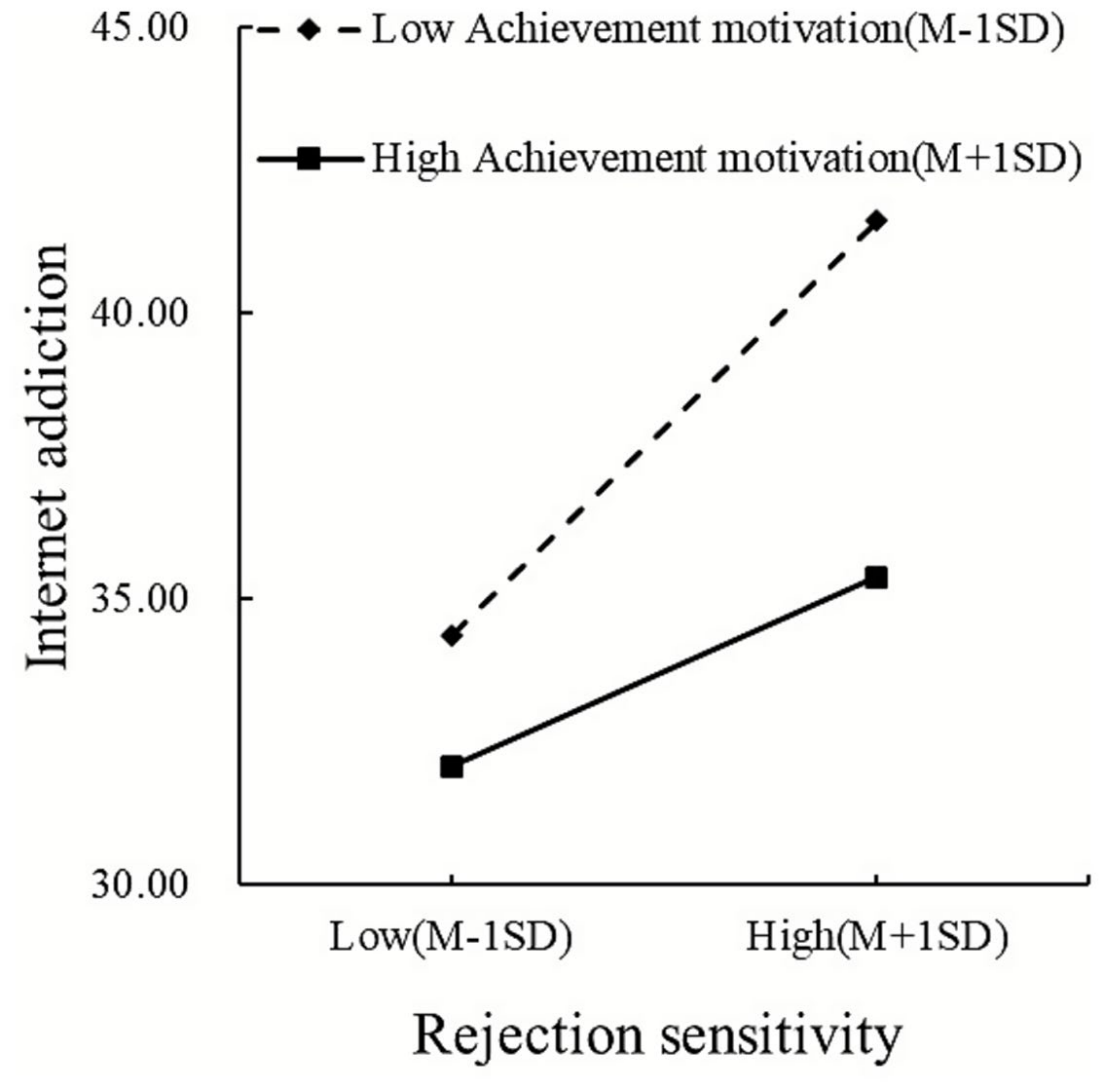
Achievement motivation moderates the rejection sensitivity–Internet addiction pathway linking authoritarian parenting to Internet addiction.

Simple slope analysis (see Figure 6) also demonstrated that effect of rejection sensitivity on IA was significantly positive at all levels but decreased as achievement motivation increased: low achievement motivation (index = 0.26, 95% CI [0.18, 0.34]), mean achievement motivation (index = 0.19, 95% CI [0.13, 0.25]), and high achievement motivation (index = 0.12, 95% CI [0.04, 0.20]).

#### 3.3.3 Indirect effects through self-control and sequential pathways

A consistent pattern emerged across all three parenting styles regarding the mediation mechanisms as shown in Table 4. Based on mean achievement motivation values, both the self-control mediation pathway and the sequential mediation pathway demonstrated significant effects for all three parenting styles. In contrast, the direct pathway through rejection sensitivity alone was non-significant across all parenting styles.

**Table 4:** Conditional indirect effects at different levels of achievement motivation and moderated mediation indices across three parenting styles (*N* = 661).

Pathway	Authoritative *B* (95% CI)	Authoritarian *B* (95% CI)	Permissive *B* (95% CI)
Self-control (M1)			
Low (−1 SD)	−0.11 [−0.18, −0.05]	0.11 [0.04, 0.20]	0.16 [0.07, 0.25]
Mean	−0.07 [−0.12, −0.03]	0.07 [0.03, 0.13]	0.10 [0.05, 0.17]
High (+1 SD)	−0.03 [−0.06, −0.002]	0.03 [0.001, 0.06]	0.04 [0.004, 0.09]
Index	0.003 [0.001, 0.01]	−0.003 [−0.006, −0.001]	−0.004 [−0.007, −0.002]
Rejection sensitivity (M2)			
Low (−1 SD)	−0.03 [−0.08, 0.01]	0.05 [−0.0004, 0.11]	−0.03 [−0.11, 0.03]
Mean	−0.02 [−0.06, 0.01]	0.03 [−0.0003, 0.07]	−0.02 [−0.07, 0.02]
High (+1 SD)	−0.01 [−0.04, 0.01]	0.02 [−0.0006, 0.05]	−0.02 [−0.05, 0.02]
Index	0.001 [−0.0003, 0.002]	−0.001 [−0.002, 0.000]	0.001 [−0.001, 0.002]
Sequential (SC → RS → IA)			
Low (−1 SD)	−0.01 [−0.03, −0.004]	0.01 [0.003, 0.03]	0.02 [0.01, 0.04]
Mean	−0.009 [−0.02, −0.002]	0.009 [0.002, 0.02]	0.01 [0.004, 0.03]
High (+1 SD)	−0.006 [−0.01, −0.001]	0.006 [0.001, 0.01]	0.01 [0.002, 0.02]
Index	0.0003 [0.0000, 0.0006]	−0.0003 [−0.0007, 0.0000]	−0.0004 [−0.0009, 0.0000]

AM, Achievement motivation; SC, Self-control; RS, Rejection sensitivity; IA, Internet addiction. Index: Moderated mediation index. Values are unstandardized conditional indirect effects at low (−1 SD), mean, and high (+1 SD) levels of achievement motivation.

Self-control demonstrated robust simple mediation for all three parenting styles, positive for authoritative parenting (index = −0.07, 95% CI [−0.12, −0.03]) versus negative for both authoritarian (index = 0.07, 95% CI [0.03, 0.13]) and permissive parenting (index = 0.10, 95% CI [0.05, 0.17]).

In contrast, the mediation pathway through rejection sensitivity alone was non-significant across all parenting styles: authoritative parenting (index = −0.02, 95% CI [−0.06, 0.009]), authoritarian (index = 0.03, 95% CI [−0.0003, 0.07]), and permissive parenting (index = −0.02, 95% CI [−0.07, 0.02]). This pattern indicates that rejection sensitivity operates primarily as a subsequent consequence of self-control deficits rather than as an independent pathway directly following parenting.

The sequential indirect effects through self-control and rejection sensitivity were also significant for all three parenting styles: authoritative parenting (index = −0.009, 95% CI [−0.02, −0.002]), authoritarian (index = 0.009, 95% CI [0.002, 0.02]), and permissive parenting (index = 0.01, 95% CI [0.004, 0.03]).

These findings indicate that self-control serves as a robust mediator linking parenting practices to IA, while rejection sensitivity operates primarily as a subsequent consequence of self-control deficits rather than as an independent mediator directly following parenting practices.

#### 3.3.4 Moderated mediation effects of achievement motivation

To test whether achievement motivation moderated the indirect effects, we examined conditional indirect effects at three levels of achievement motivation (M ± 1 SD) for each pathway. The self-control pathway exhibited significant conditional indirect effects across all levels of achievement motivation for all parenting styles (see Table 4). Notably, the moderated mediation indices revealed contrasting directional patterns: positive for authoritative parenting (index = 0.003, 95% CI [0.001, 0.01]) versus negative for both authoritarian (index = −0.003, 95% CI [−0.006, −0.001]) and permissive parenting (index = −0.004, 95% CI [−0.007, −0.002]).

Conversely, the rejection sensitivity pathway alone failed to demonstrate significant conditional indirect effects at any level of achievement motivation, with all 95% confidence intervals including zero across all three parenting styles (see Table 4).

Most importantly, the sequential pathway demonstrated significant conditional indirect effects across all levels of achievement motivation for all three parenting styles. This confirms that parenting styles influence IA through a cascading mechanism in which self-control affects rejection sensitivity, which subsequently affects IA. However, the moderated mediation indices for the sequential pathway were marginally significant or approached significance, with confidence interval boundaries touching zero: authoritative parenting (index = 0.0003, 95% CI [0.0000, 0.0006]), authoritarian parenting (index = −0.0003, 95% CI [−0.0007, 0.0000]), and permissive parenting (index = −0.0004, 95% CI [−0.0009, 0.0000]).

## 4 Discussion

The present study investigated the relationships between Baumrind’s parenting styles (authoritative, authoritarian, and permissive) and IA among Chinese primary school children, examining the mediating roles of self-control and rejection sensitivity, as well as the moderating role of achievement motivation. The findings provide important insights into the family-based mechanisms underlying children’s IA and offer evidence-based guidance for prevention and intervention strategies.

### 4.1 Parenting styles and children’s Internet addiction

Authoritative parenting’s negative association with IA, supporting Hypothesis 1a, reflects not merely high expectations but the optimal combination of structure and emotional support. This finding is particularly significant in the Chinese context, challenging traditional emphasis on strict discipline (guan) (Chao, 1994) while validating the importance of parental warmth. When parents set clear boundaries with warmth and open communication, children develop secure attachment and internalized self-regulation, reducing reliance on Internet for emotional fulfillment or escape (Monacis et al., 2017). This suggests contemporary Chinese families benefit from integrating traditional high expectations with modern emphases on emotional connection and autonomy support, aligning with Self-Determination Theory (Ryan and Deci, 2017) that environments satisfying autonomy, competence, and relatedness promote adaptive behaviors.

Conversely, authoritarian parenting’s positive association with IA, supporting Hypothesis 1b, indicates that excessive control, harsh punishment, and emotional neglect—rather than high demands per se—are associated with increased risk of problematic Internet use in children. This carries particular significance given authoritarian practices’ roots in Confucian traditions emphasizing filial piety and parental authority (Chao, 1994). However, when parents prioritize obedience over emotional connection, children experience stress, reduced autonomy, and family disconnection, making the Internet a refuge for autonomy and social connection (Kunz and Grych, 2013). This compensatory function suggests that as Chinese society modernizes, traditional authoritarian approaches may be diminishing in effectiveness, particularly where children face both traditional control and unprecedented digital freedoms (Ren et al., 2024). The psychological reactance triggered by excessive control may further lead children to engage in Internet use as rebellion (Van Petegem et al., 2015), aligning with Self-Determination Theory’s premise that environments thwarting basic psychological needs are associated with maladaptive compensatory behaviors (Ryan and Deci, 2017).

The non-significant direct effect of permissive parenting on IA contrasts with some Western research (Valcke et al., 2010) but aligns with studies reporting inconsistent or null effects (Anandari, 2016; Dogan et al., 2015), and thus failed to support Hypothesis 1c. This pattern may reflect the structurally heterogeneous nature of permissive parenting in contemporary China, where the construct encompasses qualitatively distinct subtypes with opposing influences. Specifically, “permissive parenting” in China includes both “left-behind children” experiencing involuntary parental absence due to rural-to-urban migration (Meng and Yamauchi, 2017) and children of Western-influenced urban parents who intentionally grant autonomy to foster independence (Ren and Edwards, 2015). These opposing pathways—neglect versus intentional autonomy-granting—likely cancel out at the population level, producing null aggregate direct effects. Additionally, central Chinese urban families may exhibit distinct dynamics compared to coastal metropolitan areas (Liu S.-W. et al., 2025), further contributing to regional heterogeneity. The borderline internal consistency of the permissive parenting subscale may have slightly attenuated the observed association with IA, and the near-null correlation should be interpreted with caution. Critically, permissive parenting negatively correlated with self-control, which predicted IA, indicating indirect influence. This underscores the importance of examining mediation mechanisms even when direct effects are non-significant.

### 4.2 Mediating role of self-control

Self-control emerged as a significant mediator in the relationships between all three parenting styles and children’s IA, supporting hypotheses 2a, 2b, and 2c. These findings confirm recent meta-analytic evidence establishing self-regulation as a primary protective factor against addictive behaviors (Li et al., 2021). Moreover, these results extend the strength model of self-control (Baumeister et al., 2007) to the digital domain, demonstrating that children lacking self-regulatory resources struggle to resist impulse-driven behaviors offering immediate gratification, such as gaming, social media, and video consumption (Brand et al., 2019).

Authoritative parenting predicted higher self-control, which negatively predicted IA. This finding aligns with extensive research demonstrating that supportive, structured parenting environments foster self-regulatory capabilities in children (Marcone et al., 2020). The combination of clear expectations, behavioral guidance, and positive role modeling inherent in authoritative parenting provides children with both the motivation and the skills necessary for effective self-regulation. In Chinese cultural contexts where self-regulation and behavioral discipline are highly valued (Chao, 1994), this pathway represents optimal integration of traditional “guan” (discipline) with autonomy support—children develop value-based rather than fear-based regulation, enabling resistance to Internet temptations even without parental surveillance.

Authoritarian parenting predicted lower self-control, which increased IA risk. This pathway suggests that excessive parental control may undermine children’s development of autonomous self-regulation. When children rely primarily on external punishment to control their behavior rather than developing internal regulatory mechanisms, they may struggle to resist the immediate gratification offered by Internet activities in the absence of parental surveillance. This finding extends previous research on parenting and adolescent self-control (Lei et al., 2021) to younger children, emphasizing the early importance of fostering internal regulation rather than relying solely on external control.

Notably, although permissive parenting showed no total effect on IA, it significantly predicted lower self-control, which in turn increased IA risk. This indirect-only mediation pattern reveals an important nuance: permissive parenting may undermine self-control development through insufficient guidance and structure, yet other unmeasured protective factors in permissive families (e.g., parental warmth, child autonomy) may counterbalance this risk at the total effect level. This finding demonstrates the value of examining indirect pathways even when total effects are non-significant (Preacher and Hayes, 2008) and suggests that interventions targeting self-control may be particularly relevant for children from permissive households.

### 4.3 Mediating role of rejection sensitivity

Contrary to hypotheses 3a and 3b but consistent with 3c, rejection sensitivity did not function as an independent mediator directly following parenting practices. This unexpected finding provides crucial theoretical insights into the developmental ordering of psychological mechanisms. Rather than operating as a parallel pathway alongside self-control, rejection sensitivity emerged as a subsequent consequence—a discovery with important implications for understanding IA etiology.

The pattern of results suggests that parenting practices influence rejection sensitivity primarily through their impact on self-regulatory capacities. Authoritative parenting showed no direct effect on rejection sensitivity, indicating its protective influence operates entirely through enhancing self-control. Even authoritarian parenting, which showed a significant direct path to rejection sensitivity, exerted its ultimate influence on IA through the self-control → rejection sensitivity cascade rather than through an independent direct pathway. These findings align with emerging research emphasizing executive function’s primacy in emotional development (Blair and Raver, 2015).

Recent developmental neuroscience research provides mechanistic insights into this temporal ordering. Self-regulatory capacities, grounded in prefrontal cortex maturation, develop earlier and more continuously across childhood than complex socio-emotional processing patterns like rejection sensitivity (Zelazo, 2020). Children with well-developed self-control can effectively modulate emotional responses to ambiguous social cues, reducing tendency toward anxiety-driven interpretations characteristic of high rejection sensitivity (Fan et al., 2022). Conversely, children lacking self-regulatory capacities become vulnerable to emotional dysregulation in social contexts, manifesting as heightened vigilance for rejection cues and amplified responses to perceived rejection (Shafiq and Batool, 2021).

Permissive parenting showed no association with rejection sensitivity (Hypothesis 3c). Low conflict, minimal criticism, and infrequent rejection provide fewer rejection cues than authoritarian households, not significantly impacting anticipatory anxiety. Rejection sensitivity relates more to critical or rejecting behaviors (Downey et al., 2004) than lack of structure, thus permissive parenting does not appear to be linked to this pathway to IA.

### 4.4 Sequential mediating roles of self-control and rejection sensitivity

The most significant contribution lies in demonstrating that parenting styles influence IA through an ordered developmental cascade: self-control shapes rejection sensitivity, which subsequently affects Internet use. This sequential pattern emerged consistently across all three parenting styles, supporting a developmental cascade model (Masten and Cicchetti, 2010) wherein self-control serves as the proximal mechanism directly shaped by parenting, and rejection sensitivity emerges as a distal consequence. Although this study is cross-sectional in design, the findings align with longitudinal evidence showing that early self-regulation predicts subsequent emotional reactivity, but not vice versa (Dollar et al., 2023; Spinrad et al., 2012), and extends the strength model of self-control (Baumeister et al., 2007) to developmental processes. This integrates with compensatory Internet use theory (Kardefelt-Winther, 2014): children lacking self-regulatory capacities struggle to manage emotional challenges, developing heightened rejection sensitivity that motivates escape into virtual environments. Children with both poor self-regulation and high rejection sensitivity face “double jeopardy,” explaining individual differences in IA vulnerability.

However, the directional patterns and underlying mechanisms differed meaningfully across parenting types. Authoritative parenting initiated a protective cascade: enhanced self-control reduced rejection sensitivity, ultimately decreasing IA risk. The observed mediation pattern indicates that the protective influence is mainly transmitted via psychological mechanisms. Children from authoritative homes possess both the self-regulatory capacity to resist impulsive online engagement and sufficient emotional resilience to manage offline challenges—a “double protection” eliminating the psychological need for compensatory Internet use.

In contrast, authoritarian parenting initiated a risk cascade but uniquely demonstrated both a direct effect on rejection sensitivity and an indirect effect through self-control—a “dual-pathway” mechanism. The direct pathway reflects children’s emotional responses to harsh, psychologically controlling practices, developing heightened vigilance to disapproval. The indirect pathway reflects the developmental cascade: children who fail to develop self-regulation under external control subsequently struggle to manage interpersonal challenges. Despite its emphasis on discipline, authoritarian parenting undermines internalized self-regulation by relying on external controls rather than fostering autonomous motivation (Baumrind, 1991; Steinberg, 2001). This creates “double jeopardy”: children lack both the self-regulatory capacity to resist impulsive online engagement and experience strong rejection sensitivity motivating escape into virtual environments.

Permissive parenting also initiated a risk cascade through impaired self-control and heightened rejection sensitivity but demonstrated no direct effect on rejection sensitivity—an “indirect-only” pattern indicating its influence on emotional functioning operates entirely through failing to develop self-regulatory capacities. Despite surface differences from authoritarian parenting (passive neglect versus active control), both share the critical commonality of failing to foster internalized self-regulation (Baumrind, 1991). While authoritarian parenting may actively create emotional distress (reflected in the direct path to rejection sensitivity), permissive parenting’s influence appears more passive—failing to build protective capacities rather than actively causing harm. In the Chinese context, “permissive parenting” encompasses heterogeneous subtypes—from involuntary neglect due to parental migration to intentional autonomy-granting—yet the common consequence is impaired self-regulatory development cascading into emotional vulnerabilities.

### 4.5 Moderating role of achievement motivation

#### 4.5.1 Direct moderation of mediator-outcome links

Achievement motivation significantly moderated both pathways in the sequential mediation models, supporting Hypotheses 5 and 6. These findings align with Achievement Goal Theory (Dweck and Leggett, 1988; Elliot, 1999) and the I-PACE model’s emphasis on person-specific characteristics as boundary conditions (Brand et al., 2019).

Achievement motivation attenuated the protective association between self-control and Internet addiction. Specifically, the negative relationship between self-control and IA was strongest at low levels of achievement motivation and became progressively weaker as achievement motivation increased. This pattern suggests that among children with high achievement motivation, Internet use behavior may be regulated less by effortful inhibitory self-control and more by goal-focused regulatory processes, such that long-term objectives guide behavior even when dispositional self-control is relatively low (Fujita, 2011; Milyavskaya et al., 2015). In this context, internalized achievement goals may reduce reliance on effortful self-control in guiding behavior, thereby weakening the relative influence of self-control on IA outcomes. Conversely, among children with low achievement motivation, self-control plays a more central role in constraining impulsive Internet use, such that deficits in self-control are more directly associated with higher IA risk (Duckworth and Gross, 2014). Together, these findings indicate that achievement motivation functions as a boundary condition in the sequential pathway, shaping the extent to which self-control translates into Internet addiction outcomes.

Achievement motivation buffered the maladaptive effects of rejection sensitivity on IA, such that high achievement motivation weakened this positive association while low achievement motivation amplified it. Achievement-oriented children’s mastery focus and future orientation provide psychological resilience, anchoring self-worth in goal progress rather than social acceptance (Dweck, 2006; Duckworth et al., 2007). When facing rejection cues, these children maintain engagement in constructive activities rather than withdrawing into excessive online engagement. Conversely, rejection-sensitive children with low achievement motivation lack these protective resources and become particularly susceptible to using the Internet as a means of avoiding rejection anxiety (Kardefelt-Winther, 2014), creating a “double vulnerability” that substantially amplifies IA risk.

#### 4.5.2 Moderated mediation: parenting style-specific patterns

Achievement motivation significantly moderated the self-control pathway to IA across all three parenting styles, consistently attenuating the indirect effect of parenting on IA through self-control. Specifically, as achievement motivation increased, the mediating role of self-control in the parenting–Internet addiction association became weaker.

This pattern is consistent with goal-based models of self-regulation, which suggest that internalized achievement goals can guide behavior through goal-oriented processes, thereby reducing reliance on effortful self-control (Fujita, 2011; Milyavskaya et al., 2015). From a self-determination theory perspective (Ryan and Deci, 2017), authoritative parenting is more likely to foster autonomous achievement motivation, allowing goal regulation to partially substitute for self-control in shaping Internet use behavior. In contrast, under authoritarian and permissive parenting, where motivational regulation may be less autonomous or less consistently supported, self-control remains a more central mechanism linking parenting experiences to Internet addiction risk. Together, these findings suggest that achievement motivation functions as a boundary condition across parenting styles, shaping the extent to which self-control mediates the association between parenting and Internet addiction.

Although achievement motivation moderated the direct link from rejection sensitivity to IA, it did not produce a significant moderated mediation effect through rejection sensitivity alone across the three parenting styles. This indicates that while motivational resources can buffer behavioral outcomes once self-regulatory or emotional vulnerabilities are triggered, they do not alter the earlier family-based mechanisms through which such vulnerabilities are formed. Rejection sensitivity may thus act primarily as a subsequent affective manifestation of self-control deficits rather than as an independent mediating mechanism directly following parenting. From a dual-systems perspective (Shulman et al., 2016; Telzer, 2016), this pattern suggests that “hot” emotional processes are less amenable to regulation by “cold” cognitive–motivational systems, especially during late childhood when emotion regulation remains immature (Schweizer et al., 2020).

Notably, while achievement motivation consistently moderated the self-control pathway, evidence for moderation of the full sequential pathway (parenting → self-control → rejection sensitivity → IA) was inconclusive—the indices of moderated mediation included zero in their confidence intervals across all three parenting styles. This pattern tentatively suggests that achievement motivation may play a more prominent role in shaping the association between self-control and IA, rather than in moderating the link between rejection sensitivity and IA. The stage-specific nature of this pattern has practical implications: interventions targeting self-control may benefit from incorporating motivational components, whereas the pathway through rejection sensitivity appears less influenced by achievement motivation. This also implies that once emotional vulnerabilities such as rejection sensitivity are established, motivational enhancement alone may provide limited additional benefit (Yeager et al., 2019; Pfeifer and Allen, 2021).

### 4.6 Practical implications

The findings offer several practical implications for preventing children’s IA. First, parent education programs focusing on authoritative parenting may be promising targets for intervention (Theopilus et al., 2024; Wang et al., 2024), which integrates clear behavioral expectations with emotional warmth and responsiveness. A recent systematic review of preventive interventions found that parenting strategy approaches showed promising efficacy in reducing IA risks with small-to-medium effect sizes (Theopilus et al., 2024). Specifically, parents are encouraged to establish consistent rules for Internet use, explain the rationale behind these rules, encourage open dialogue about online activities, and provide emotional support when children encounter difficulties (Theopilus et al., 2024).

For authoritarian families, interventions could emphasize the integration of discipline and warmth, consistent with the Chinese concept of “guan” (governance with affection) (Wu, 2012). Simply imposing rules without emotional responsiveness may hinder children’s ability to internalize behavioral norms (Grusec and Goodnow, 1994; Li et al., 2023). In contrast, providing warmth and support alongside clear expectations helps children develop self-control (Wang et al., 2007; Li et al., 2023), which in turn may reduce rejection sensitivity and lower the risk of IA (Rohner, 2017; Rothenberg et al., 2022). For permissive families, guidance should consider the cultural and structural realities of Chinese society. Evidence from community-based interventions suggests that in left-behind or migrant families, community-based supervision may compensate for limited parental monitoring (Zhao et al., 2017; Hesketh et al., 2020; Zhang et al., 2020). For instance, a study of community-based children’s centers in rural China demonstrated improvements in psychosocial well-being among left-behind children, particularly in areas where homework supervision and structured activities were provided (Hesketh et al., 2020). In urban families influenced by liberal parenting beliefs, education programs could highlight the importance of structure and routine in fostering children’s self-control within a supportive family environment (Maccoby and Martin, 1983; Li et al., 2023; Li et al., 2025).

Second, interventions could target self-control as a key mechanism. Evidence from mindfulness-based and self-regulation training programs have been shown to enhance children’s attention, impulse control, and emotion-regulation capacities (Bockmann and Yu, 2023; Lawler et al., 2019; Porter et al., 2024). Such programs may also help children reinterpret rejection experiences and develop healthier emotional responses. Moreover, given the sequential mediation pathway identified in this study, early intervention focusing on parenting and self-control may prevent the cascade toward heightened rejection sensitivity and IA. Consistent with the developmental cascade framework (Masten and Cicchetti, 2010), “breaking the chain” during early childhood—when self-regulatory capacities are still forming and family influences are most salient—could yield more enduring protective effects.

Third, fostering achievement motivation may serve as a protective buffer. Educational programs helping children set meaningful goals and develop mastery experiences can enhance resilience against both low self-control and rejection sensitivity. However, healthy achievement motivation should be distinguished from excessive performance pressure, which may be associated with increased Internet use as an escape in children.

Finally, tailored, multi-level intervention strategies are needed. School-based universal prevention programs that integrate parent training, children’s self-regulation skill development, and institutional support may be most effective in reducing IA risk across diverse family contexts, complementing family-focused interventions.

### 4.7 Limitations and future directions

Several limitations should be acknowledged. First, the cross-sectional design substantially limits causal inference and prevents examination of temporal or developmental relationships among parenting styles, self-control, rejection sensitivity, and IA, as these constructs are likely to change across childhood development. Longitudinal or experimental studies are needed to clarify causal pathways and test developmental trajectories. Second, all variables were assessed via child self-report in a single session, which raises concerns regarding common method bias and social desirability effects. Although Harman’s single-factor test did not indicate a single dominant factor, other sources of method bias cannot be fully ruled out. Procedural safeguards—including confidentiality, standardized instructions, and exclusion of inattentive responses—were implemented to reduce bias, but future research should incorporate multi-informant assessments (e.g., parent reports of parenting and children’s IA, teacher reports of self-control), behavioral observations, and objective measures of Internet use to enhance measurement validity.

Third, listwise deletion was used to handle missing data, which may introduce bias if data are not missing completely at random (MCAR). Although the proportion of missing data was small, future studies should consider alternative approaches such as multiple imputation or full information maximum likelihood (FIML) to address potential biases associated with missing data. Fourth, the permissive parenting subscale demonstrated borderline internal consistency, which may have attenuated correlations with IA and contributed to the near-null association observed. Accordingly, findings related to permissive parenting should be interpreted with caution. In addition, although confirmatory factor analyses were conducted for all measures, some fit indices (CFI/TLI) fell below the commonly recommended threshold of 0.90. Specifically, the Western-developed parenting measure showed suboptimal fit, which is consistent with prior findings that such instruments may not fully capture the nuances of parenting in Chinese cultural contexts. Similarly, the Grasmick Self-Control Scale demonstrated high internal consistency but suboptimal factor structure, possibly because it was originally developed for adults rather than children. While these measurement limitations did not affect our primary analyses—which were based on composite scores with acceptable internal consistency rather than latent variable modeling—future studies should develop and validate culturally appropriate parenting measures and age-appropriate self-control instruments for Chinese primary school populations.

Fifth, the study sampled children from two urban public schools in central China, which may limit generalizability to regions with substantially higher or lower socioeconomic levels. Given China’s substantial regional variation in economic development and educational philosophies, the identified patterns may differ in highly developed metropolitan areas or less developed regions. Future research should examine whether these findings replicate across regions with diverse economic and cultural contexts. Sixth, the present study did not distinguish between specific types of Internet use or examine potential gender differences in the proposed pathways. Not all Internet activities are equally problematic, as educational use may differ substantially from gaming or social media engagement. Moreover, boys and girls may respond differently to parenting practices and exhibit distinct Internet use patterns. Although preliminary analyses suggested no significant gender differences in the key study variables, future studies with larger samples are warranted to investigate type-specific and gender-specific pathways. Finally, the study did not assess how children’s characteristics may influence parenting behaviors. Transactional models suggest that parent–child influences are reciprocal, with children’s IA, self-control, or rejection sensitivity potentially eliciting different parenting responses. Future research employing cross-lagged panel designs could examine these bidirectional relationships.

## 5 Conclusion

This study provides comprehensive evidence that authoritative, authoritarian, and permissive parenting styles all influence children’s IA through the sequential mediating pathways of self-control and rejection sensitivity, with achievement motivation serving as a significant moderator. Authoritative parenting protects against IA by enhancing self-control and then reducing rejection sensitivity, whereas authoritarian and permissive parenting increase addiction risk through the opposite pathways. Achievement motivation serves as a contextual protective factor, weakening the negative association between self-control and IA as well as the positive association between rejection sensitivity and IA.

These findings highlight the importance of family-based prevention strategies that promote authoritative parenting while simultaneously fostering children’s self-control, emotional regulation, and achievement motivation. By elucidating the complex psychological processes linking parenting to children’s IA, this study offers both theoretical insight and practical guidance for developing culturally sensitive, evidence-based interventions to address the growing public health concern of IA among Chinese children.

## Data Availability

The raw data supporting the conclusions of this article will be made available by the authors without undue reservation.
