# Supplementary figures and images for: Parenting styles and Internet addiction in Chinese primary school students: a moderated sequential mediation model of self-control, rejection sensitivity, and achievement motivation
Source: Front Psychol. 2026 Jan 29;17:1742553. doi: 10.3389/fpsyg.2026.1742553 (PMC12896228; doi:10.3389/fpsyg.2026.1742553)

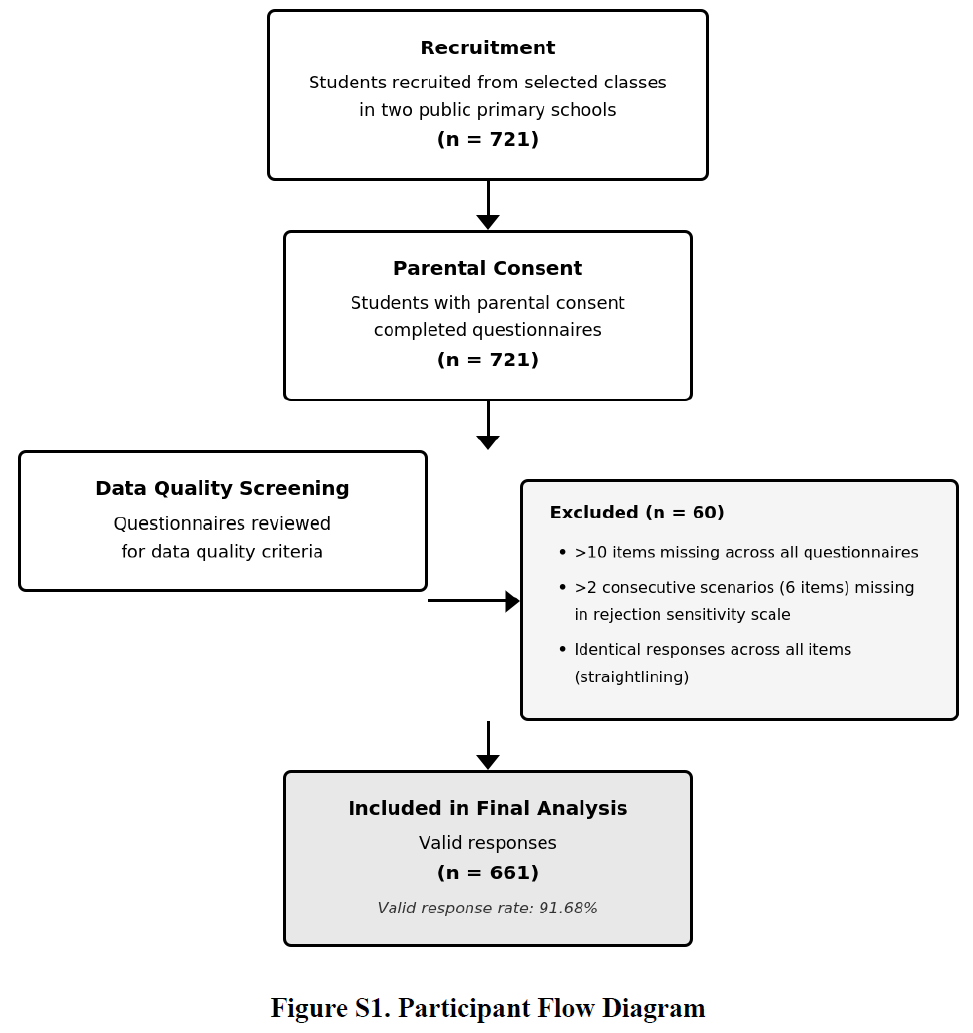

Supplement: Supplementary file 1 [file Image_1.TIF]
